# Associations Among Maternal Positivity, Negativity and Child Attachment in the Netherlands, Poland, and Turkey

**DOI:** 10.3389/fpsyg.2022.820699

**Published:** 2022-05-17

**Authors:** Katarzyna Lubiewska, Nebi Sümer, Karolina Głogowska, Özlü Aran, Wouter de Raad

**Affiliations:** ^1^Department of Psychology, University of Warsaw, Warsaw, Poland; ^2^Department of Psychology, Kazimierz Wielki University, Bydgoszcz, Poland; ^3^Faculty of Arts and Social Sciences, Sabanci University, Istanbul, Turkey; ^4^Department of Psychology, University of Denver, Denver, CO, United States

**Keywords:** attachment, cross-cultural comparisons, parenting, Middle childhood, culture fit

## Abstract

This study addresses how maternal positivity and negativity toward a child in three countries, separately and in combination are related to attachment in middle childhood. We first developed an ecologically valid emic measure of the Maternal Positivity-Negativity Scale through an interview-based study (90 mothers) and then tested our hypotheses in a separate study. The child’s attachment security (where the child uses the mother as a safe haven and secure base) and insecurity (attachment anxiety and avoidance) were assessed using standard measures. Equal numbers of mothers and their children between 8 and 12 years of age from Poland, Turkey, and the Netherlands participated in the main study (756 dyads). Results revealed that: (1) maternal positivity was more strongly associated, than maternal negativity, with child security; (2) maternal negativity was more strongly associated, than maternal positivity, with child anxiety, and its relation was stronger when maternal positivity was low; (3) maternal negativity was more strongly associated with child anxiety than with child avoidance; (4) the maternal positivity-over-negativity prevalence index was related to child attachment security and insecurity; (5) relations between maternal positivity and child attachment were moderated by culture. Results are discussed considering attachment in middle childhood and culture-related perspectives.

## Introduction

Attachment security develops in infancy and is delineated by the trust that a caregiver, usually a parent, is responsive and available to provide a *safe haven* in times of distress and a *secure base* for exploration in non-threatening conditions (Ainsworth et al., 1978/2015). If, however, a parent is perceived as unavailable, children develop insecure adaptational strategies. They increase their monitoring of the availability of a caregiver and may become hypervigilant to signals of potential parental unavailability or lack of parental acceptance. These attachment-related concerns may be dealt with by maintaining attachment anxiety and proximity seeking efforts in child-to-parent relations (attachment anxiety) or by defensive withdrawal and avoiding proximity with a parent to handle threat alone (attachment avoidance) (Bowlby, 1980).

In studies assessing attachment in middle childhood and beyond with the use of self-report scales, low levels of attachment anxiety and attachment avoidance are treated as attachment security indicators (Brennan et al., 1998). Therefore attachment security and insecurity indicators are rarely studied together. However, there is an evidence that attachment anxiety and attachment avoidance scales do not measure attachment security well (Fraley et al., 2000) and thus scholars suggest they be studied in combination (Brenning et al., 2011).

A caregiver’s sensitive and responsive parenting is an established precursor of infant attachment security (e.g., Fearon and Belsky, 2016). However, sensitivity includes multiple parenting constructs and measures (De Wolff and van Ijzendoorn, 1997; Mesman and Emmen, 2013; Koehn and Kerns, 2018; O’Neill et al., 2021), thus may be approached from different perspectives. Furthermore, the expression, as well as the child’s interpretation of parenting behaviors as more or less sensitive is related to various factors, like parental or child temperamental characteristics or culture. The effect of cultural context on relations between parenting and attachment is rarely studied, though. Thus, studies on associations between parenting and child attachment, as well as studies on effects of culture on parenting—attachment relations are needed.

### Parenting and Child Attachment

Although studies in middle childhood are scant, a meta-analysis by Koehn and Kerns (2018) on multiple parenting constructs evidences that mothers of secure children are supportive and predictable, whereas mothers of insecure children tend to be intrusive and overstimulating (avoidant children) or inconsistent and unpredictable (anxious children). These findings indicate that the emotional tone of the parent-child relationship, associated with parental positivity (supportiveness) and negativity (intrusiveness and overstimulation), alongside predictability of parental behaviors, may foster or undermine child attachment security.

Parental positivity and negativity are broadly and inconsistently defined in previous studies by: negative/positive feelings (Knafo and Plomin, 2006) and their observable (Eisenberg et al., 2005), verbal or physical (Meunier et al., 2011) expressions; conflict/warmth (Feinberg and Hetherington, 2001), and hostility, rejection (Gölcük and Berument, 2021); negative/positive discipline (Knafo and Plomin, 2006), including reinforcements/punishment (Booster et al., 2016); and unclear directives/clear rules (Booster et al., 2016). Positivity and negativity definitions cover a broad range of, more or less, related parenting dimensions treated as parental sensitivity indicators (De Wolff and van Ijzendoorn, 1997).

In the present study, we defined parental positivity and negativity broadly as a range of parental behaviors undertaken by parents to express their warmth, attention, or withdrawal of warmth, hostility, or demandingness toward their own children in verbal or non-verbal forms. However, we also propose to ask parents how they express positivity and negativity toward their children beyond standardized parenting scales. This approach might be helpful in better understanding the positivity-negativity construct in an ecologically valid way. Moreover, approaching parenting from a perspective of parental positivity and negativity expressed to the child may be valuable for clinical practice. Experiences of individuals about whether their parents were more positive or negative toward them in their childhood are reflected in their mental representations of their own parents. Through this lens children (Shmueli-Goetz et al., 2008) and adults (Hesse, 2008) discuss their childhood memories in attachment-based interviews and choose adjectives they use to describe their own parents. These interview-based assessment methods have a high status in attachment research and practice. Thus, the proposed perspective on parenting quality, especially when parental positivity and negativity are analyzed from the perspective of parents (rather than from the angle of standardized parenting scales) may bring analyses of parenting quality closer to individual experiences.

Analysis of parenting through the lens of parenting positivity and negativity leads to an important question for parenting practices: What is a stronger predictor of child attachment security and insecurity—the daily expressions of maternal positivity, the daily expressions of maternal negativity, or the combined effect of maternal positivity and negativity? Answering this question, above and beyond the assessment of the most frequently studied parenting dimensions (e.g., warmth, control or rejection), may shed new light on the correlates of attachment in middle childhood. However, to answer this question, parenting positivity and negativity have to be analyzed as separate but also combined factors associated with child attachment.

#### Specific, Competitive and Combined Effects of Maternal Positivity and Negativity on the Child’s Attachment

Parental negativity, as associated with overt rejection, prohibiting, conditional regard, or control, is related to adverse developmental outcomes in children (Dunn et al., 1998; Deater-Deckard et al., 2006), including attachment insecurity (Swanson and Mallinckrodt, 2010; Vansteenkiste et al., 2014; Ainsworth et al., 1978/2015). However, infant attachment studies that have focused on parental positivity show more mixed findings. While some studies document an association between positivity and child attachment security (Nievar and Becker, 2008; Matias et al., 2014; Bailey et al., 2016), other studies evidence a relationship between parental positivity and child insecurity (Maximo et al., 2011) or reveal null findings (Cyr et al., 2014; Matias et al., 2014). These inconsistencies in research results may indicate that the relationship between parental positivity and child attachment may be more sensitive to contextual factors than maternal negativity as reflected in characteristics of the varying samples utilized in the previous studies.

##### Competitive Effects of Parenting Positivity and Negativity

Some studies suggest that the effects of maternal negativity may be stronger than the effects of maternal positivity on child attachment. In a meta-analytic paper titled “Bad is stronger than good,” Baumeister et al. (2001) suggest that developmentally adverse (negative) parental behaviors may influence child developmental outcomes more strongly than developmentally favorable (positive) parental behaviors. An evolutionary perspective supports this notion stressing that survival requires urgent attention to possible bad outcomes, but it is less urgent with regard to good outcomes (Baumeister et al., 2001). Studies testing the diathesis-stress model provide additional support suggesting that children who are genetically more vulnerable to environmental influences are more susceptible to negative but not to positive maternal parenting behaviors (Barry et al., 2008). The premise that negativity has a stronger effect than positivity on child attachment also has some support in attachment theory (as discussed by Berlin et al., 2008).

##### The Interplay Between Parenting Positivity and Negativity

The combination of maternal positivity and negativity can be studied in at least two ways. One way is to analyze the weight of maternal positivity over maternal negativity (or vice versa). This analysis informs about the prevailing, positive or negative climate in the relationship between the mother and the child (which we refer to as the positivity-to-negativity ratio). The second way is to analyze the interaction between the maternal positivity and negativity toward the child, which informs about whether a specific configuration of maternal positivity and negativity levels differently relate to child attachment than the other positivity-negativity configuration (which we refer to as the positivity-negativity interaction).

In the balance theory of marriage, Gottman and Levenson (2000) revealed that the quantitative advantage of negativity over positivity in intimate couples predicts a relationship breakdown with approximately 90% accuracy. Building on the balance theory of marriage, Zemp et al. (2019) show that children from families characterized by a low positivity-very high negativity ratio in inter-parental communication were more likely to have internalizing problems. The buffering effect of parental positivity on the relationship between parental negativity and a child’s internalizing problems has also been found in other studies (Kochanska et al., 2009; Measelle and Ablow, 2017; Oliver and Pike, 2018). Even though similar evidence in the attachment context is lacking, it seems likely that maternal negativity may have stronger adverse effects on child attachment in mother-child relationships where maternal positivity is low.

### Effects of Culture on the Relations Between Parenting and Child Attachment

Parents might intentionally engage in positive or negative behavior to control the child’s psychology or behavior or to convey parental warmth (or rejection). Additionally, children may interpret parenting behaviors differently in various cultural settings. Consequently, child-related developmental outcomes of parental use of positivity, negativity, and their combination may vary across cultural contexts (Pomerantz and Wang, 2009).

According to the *culture fit hypothesis* (Friedman et al., 2010), a child’s perception of being parented in the way that fits into one’s own culture (parenting-culture fit conditions) should hypothetically result in heightened satisfaction and a sense of adaptation for the child, while a lack of cultural fit (parenting-culture misfit conditions) is usually linked to distress, anxiety, and the risk of negative social evaluations. Thus, even though maternal negativity is developmentally aversive and maternal positivity is developmentally beneficial, the culture fit hypothesis suggests that associations of parental positivity and negativity with child attachment dimensions may be additionally attenuated (positivity/negativity-culture fit) or amplified (positivity/negativity-culture misfit) by culture. The logic behind the culture fit hypothesis as applied to parent-child relations must target two criteria: developmentally beneficial vs. developmentally aversive and normative vs. non-normative behaviors in any given culture.

A few studies support the culture fit hypothesis (Lansford et al., 2005; Güngor and Bornstein, 2010). To give one example, Güngor and Bornstein (2010) revealed that paternal psychological control was associated with adolescents’ attachment avoidance in Belgium but not in Turkey where parental control is more normative. It should be noted that studies testing cultural variations in both, attachment-related developmental outcomes and parental positivity are lacking. It is possible, yet not evidenced, that while positive and culturally normative parental behaviors will have a weaker positive effect on child attachment, culturally non-normative but positive parental behaviors will have a stronger positive effect. Thus, it is unknown how maternal positivity (possibly sensitive to cultural influences) not fitting cultural expectations, will be related to child attachment security.

Cultural contexts of socialization in Poland, Turkey, and the Netherlands seem to foster parental negativity and positivity differently. The traditional model of socialization in Poland is based on criticism, parental control, fostering independence, restraint, and explicit expression of negativity (Wojciszke, 2004; Lubiewska, 2019). Thus, it seems that in the Polish cultural context traditional negativity prevails over positivity in every day parent-to-child relations. Yet, parental positivity appears as a recent novel and socially promoted way of parenting. The Polish context can be contrasted by more indulgent socialization contexts in the Netherlands and Turkey, where parental warmth expression in parent-to-child relations is traditionally more fostered in socialization (Delaney, 2010). Both contexts differ regarding independent (Dutch) and interdependent (Turkish) value-orientation, stronger tightness of social norms in Turkey (Uz, 2014), and the role of psychological control, more accepted in the Turkish than the Dutch cultural context. Thus, both positivity and negativity in parent-to-child relations may be culturally normative in Turkey, whereas mainly positivity in the Dutch context.

### General Aim of the Study

Our study aims to test the extent to which child attachment is related to maternal positivity and maternal negativity as well as to their combination in three different countries in which maternal positivity and negativity are assumed to be more or less normative. To this end and in light of the variety of instruments used to assess parental positivity and negativity, as well as aiming to capture maternal positivity and negativity expressions from the perspective of parents in a culture-sensitive way, we developed an emic maternal positivity-negativity scale in the preliminary qualitative study. Next, we used this emic scale, resulting in the qualitative study, first, to test its psychometric properties and convergent validity, and then to test our main parenting-attachment hypotheses in the main quantitative study.

## Preliminary Qualitative Study

Previous studies offer a broad definition of the constructs of parental positivity and negativity based on the instruments they used. Furthermore, the cultural validity of these instruments was not addressed in the context of maternal positivity and negativity expression before. Thus, in the preliminary qualitative study, we aimed to develop an emic Maternal Positivity-Negativity scale in three national samples of mothers of middle aged children in the Netherlands, Poland, and Turkey.

### Participants

A sample of 90 mothers native to their own countries participated in interviews and an additional sample of 54 mothers participated in focus group meetings (30 and 18 mothers in each country, respectively). Mothers were between the ages of 26 and 52 (*M*_*age*_ = 39.04, *SD*_*age*_ = 6.61) with at least one child aged between 8 and 12 (*M*_*age*_ = 9.52, *SD*_*age*_ = 1.59). Data was collected between September 2017 and January 2018. The education level of interviewed mothers was diversified in each country, where eight female respondents had basic vocational education (or its equivalent in a given country), 10 secondary, and 12 higher education. Overall, 60 mothers were working, and 60% had full-time jobs. Also, 60% of mothers lived in urban areas.

### Procedure

The present study is part of the combination of emic and etic approach to parenting and attachment (CEE-PaAtt) project^1^ aiming to study attachment-related parenting of mothers from the (emic) perspective of the Dutch, Polish, and Turkish mothers and their children. Individual In-Depth Interviews (IDIs) were conducted by interviewers trained by primary researchers in each country. Interviews with mothers were carried out in focus group meeting rooms in their national languages. Mothers were randomly recruited in each country by professional companies experienced in sampling for social surveys in the qualitative study. Families were invited to a focus group meeting and asked to fill in a set of the same questionnaires. Respondents received 20 Euros each for participating in the study. All persons gave their informed consent prior to their inclusion in the study. The study was approved by the ethics committee of Kazimierz Wielki University.

Two focus group sessions (FGs) preceding and following IDIs were carried out with 18 mothers (not participating in IDIs) in each country. Mothers were asked about: (1) their parenting behaviors in various situations related to activation of attachment behavioral system of their child (e.g., child’s distress or exploration); and (2) culture-specific parenting behaviors normative in their own culture/country. FGs provided a list of attachment related aspects of maternal parenting addressed by mothers. These parenting aspects were used as the basis for semi-structured interview’s questions about attachment-related parenting and covered the following topics: maternal positivity and negativity expression, criticizing, complementing, control, overprotection vs. distanced parenting, and parenting/caregiving in five situations introduced to mothers in vignettes (child-related distress situation, child exploration, maternal availability in a socially difficult situation, child’s misbehavior, child’s exploration, and maternal praising) (see also Lubiewska et al., in review).

The interview started with a short introduction to the study and was followed by demographic questions (the child’s age, siblings, other caregivers, and maternal work and education status). Then, mothers were asked semi-structured questions about parenting, including “*How do you express positive/negative feelings toward your child?.*” Mothers were asked to speak freely. Interviewers gave prompts only in cases where the clarity of the narration was lacking or to get information about concrete (rather than general) maternal behaviors. The interviews were audio-recorded, transcribed verbatim, and translated into English.

The conceptual content analysis of interviews with blind coding was conducted twice by two national collaborators in each country and afterward in the cross-cultural group. Parenting behaviors found by each coder were compared (if necessary) and discussed until an agreement between coders was achieved regarding the formulation of the name of the parenting behavior in question, first within and then across cultural groups.

### Results of the Preliminary Qualitative Study

The topic of parental positivity and negativity expression was raised by mothers during FGs and discussed briefly by mothers in interviews where they were asked the question “*How do you express positive/negative feelings toward your child?*”

Full agreement was achieved between coders revealing that mothers in all groups reported to express their positivity by: (1) hugging; (2) kissing; (3) stroking head or ruffling the hair of their child; (4) love verbalization; (5) spending time together; (6) offering the child something they like; (7) showing more warmth in some other way. Negativity was reported as shown by: (1) saying how angry the mother is; (2) saying how disappointed mother is; (3) saying what mother thinks and feels; (4) not talking to the child; (5) paying no attention to the child; (6) being offended; (7) raising voice or shouting; (8) prohibiting things; (9) pinching or smacking; (10) punishing the children in some other way.

Results of the frequency analysis (Supplementary Material A: Table 1) revealed that most of mothers were kissing their children to express their positive feelings. Hugging or cuddling and spending time together were less common in the group of Turkish than in the groups of Polish and Dutch mothers. Ruffling hair or stroking the child’s head and showing positivity in another way (e.g., by saying how much they do for their child) was relatively more frequent in Turkish group than in other groups of mothers. The Polish mothers were giving more attention than other mothers to their child, whereas the Dutch mothers were spending more time than other mothers together with the child, verbalizing love, and cooking or giving treats to their children.

**TABLE 1:** Demographic characteristics of the sample.

Sample characteristics	Total sample	Poland	The Netherlands	Turkey	*F*/χ*^2^*
Sample size	758	258	250	250	−
**Child characteristics**					
Age (*SD*)	10 (1.4)	10 (1.4)	10 (1.3)	10 (1.4)	0.027
Sex: Female	52.2%	58.1%	48.4%	50%	5.57
Birth order: First	61.3%	61.2%	51.2	71.6%	21.94***
**Maternal characteristics**					
Age (*SD*)	38.5 (5.5)	38.1 (5.6)	41.5 (4.3)	35.8 (5.1)	79.65***
Education level					275.25***
Lower	32.6%	8.9%	31.2%	58.4%	−
Secondary	38.4%	53.9%	35.6%	25.2%	−
Higher	29%	37.2%	33.2%	16.4%	−
Urban living	64.1%	57.4%	64%	70%	23.53*

Education level: Lower—completed primary school or lower (9 or less years of education); Secondary—completed vocational or high school; Higher—completed bachelor’s degree or higher. *p < 0.05; ***p < 0.001.

Maternal negativity was expressed by the Dutch mothers mainly through verbalization of maternal feelings and thoughts but also by the withdrawal of mother-to-child directed verbalization. The Polish mothers were mainly raising their voice or shouting, prohibiting, and as well as the Turkish mothers being offended at the child. The Turkish mothers were also prohibiting things in other ways (e.g., by threatening the child that the mother will get ill, will have her heart broken, will throw a slipper at the child). Based on the results of the qualitative study the Maternal Positivity-Negativity scale (Supplementary Material A: Table 2) was developed and used in the Main Study.

**TABLE 2:** Chi square differences (χ^2^_*diff*_) of: (1) a model with free estimated paths versus (2) a model with two compared paths set equal (positivity-attachment versus negativity-attachment; anxiety-negativity/positivity versus avoidance-negativity/positivity; and safe haven-negativity/positivity versus secure base-negativity/positivity) in cultural groups and in the total sample.

Endogenous latent variable	χ^2^_*diff*_ after beta weights set equal			
	Poland	Turkey	Netherlands	Total sample
**Hypothesis 1: Positivity (P) vs. Negativity (N)**				
Anxiety	2.446 (P = N)	5.011* (P < N)	0.203 (P = N)	4.558* (P < N)
Avoidance	12.155*** (P > N)	0.371 (P = N)	0.290 (P = N)	2.542 (P = N)
Safe haven	13.923*** (P > N)	1.046 (P = N)	1.622 (P = N)	8.364** (P > N)
Secure base	9.157** (P > N)	1.438 (P = N)	0.556 (P = N)	6.076* (P > N)
**Hypothesis 2: Anxiety (Ax) vs. Avoidance (Av)**				
Negativity	4.492* (Ax > Av)	6.661** (Ax > Av)	5.061* (Ax > Av)	9.885** (Ax > Av)
Positivity	1.729 (Av = Ax)	6.166* (Ax < Av)	1.340 (Av = Ax)	9.464** (Ax < Av)
**Safe haven (SH) vs. Secure base (SB)**				
Negativity	0.295 (SH = SB)	0.237 (SH = SB)	0.005 (SH = SB)	0.221 (SH = SB)
Positivity	0.248 (SH = SB)	0.197 (SH = SB)	1.970 (SH = SB)	0.173 (SH = SB)

*p < 0.05; **p < 0.01; ***p < 0.001.

### Discussion of the Preliminary Qualitative Study

Maternal behaviors provided by the Dutch, Polish and Turkish mothers in our qualitative study overlap with the positivity-negativity conceptualization proposed in the present study defining parental positivity and negativity through child-directed parental behaviors undertaken by mothers to express their warmth, withdrawal of attention or warmth, hostility or demandingness toward their own children in a verbal or non-verbal way. These behaviors cover two aspects of parental positivity and negativity expression. First, parenting positivity-negativity behaviors found in our study address various forms (shouting, raising voice) and content (feelings and thoughts verbalization) of verbal positivity-negativity expression. Secondly, positivity-negativity behaviors of mothers address non-verbal maternal parenting positivity and negativity expression by increased (e.g., indulging the child with physical closeness, gifts and offering treats) or withhold (e.g., being offended, not talking) maternal involvement in child’s activities. These maternal behaviors were discussed by mothers in the context of parenting warmth and demandingness also analyzed in the previous studies (Feinberg and Hetherington, 2001; Knafo and Plomin, 2006; Booster et al., 2016; Gölcük and Berument, 2021). Yet, it is worth noting that maternal behaviors found in our study, in majority, do not overlap with standardized well-known parenting measures suggesting the cross-cultural fidelity of our instrument. Thus, our results preliminarily indicate that the novel positivity-negativity measure may be more ecologically valid than instruments used in previous studies in the context of Poland, the Netherlands, and Turkey.

## The Main Quantitative Study

Two goals were formulated in the main quantitative study. First, we aimed to validate the novel emic measure developed in the preliminary qualitative study to assess maternal positivity and negativity with regard to its psychometric properties and convergent validity with other well-known parenting and mother-child relationship quality measures. Second, and most importantly in our study, we aimed to test relations between child attachment and maternal positivity, negativity and their combination in three cultural groups of mother-child dyads.

### Hypotheses

Although attachment anxiety, avoidance, safe haven, and secure base are conceptually different constructs, there is a lack of literature testing these constructs in one study. Therefore, by including these constructs and testing positive parenting behaviors based on the culture fit hypothesis, our study offers a novel and thorough understanding of positive parenting and attachment behaviors. However, a lack of data about these measures limits the precision of hypotheses in our study. Based on Koehn and Kerns’s (2018) meta-analysis, we developed only one specific avoidance-related hypothesis, while leaving our other hypotheses addressing child attachment security (safe haven and secure base) and insecurity (anxiety and avoidance) at the explorative level.

First, we hypothesize that maternal negativity will be more strongly (positively or negatively) related to all child attachment dimensions than maternal positivity (*Hypothesis 1*). Second, we hypothesize that maternal negativity will be more strongly and positively related to children’s attachment avoidance than attachment anxiety (*Hypothesis 2*). This expectation was based on two premises: (1) different emotional/behavioral basis of attachment anxiety and avoidance; and (2) the results of the meta-analysis (Koehn and Kerns, 2018) suggesting a link between parental intrusiveness, conceptually similar to our negativity measure, and attachment avoidance. Third, we hypothesize that the greater ratio of maternal positivity over negativity (which we refer to as the positivity-to-negativity ratio) toward the child will be positively related to child attachment security and negatively related to child attachment insecurity (*Hypothesis 3*). Fourth, we hypothesized that the positive associations between maternal negativity and child attachment insecurity dimensions will be stronger when the level of maternal positivity is low (*Hypothesis 4*). Testing the third hypothesis, we will answer the question of whether the ratio of greater maternal positivity over negativity (or vice versa) is associated with child attachment dimensions to a greater extent than maternal positivity and negativity in separation. Testing the fourth hypothesis, however, we will analyze the interactive (not prevalent) roles of positivity and negativity in relation to child attachment. Results of both hypotheses testing will inform psychological practice regarding whether attachment-related effects of parenting interventions may be optimal when the intervention is based on increasing maternal positivity or decreasing maternal negativity (Hypotheses 1–3) and whether maternal positivity may effectively buffer the negative effect of maternal negativity on child attachment (Hypothesis 4).

Addressing cross cultural differences in maternal-child relations, we hypothesized that: the negative relationship between maternal negativity and child attachment security will be stronger in the Dutch sample (negativity-culture misfit) compared to the Polish and Turkish samples (*Hypothesis 5*); and maternal positivity, as well as a ratio of positivity-over-negativity prevalence will be more strongly and positively related to child attachment security in the Polish sample (positivity-culture misfit) than in the Dutch and Turkish samples (*Hypothesis 6*).

Even though the scale validation is one of our aims in the main quantitative study, the scale is implemented further in this study to test relations between child attachment and maternal positivity and negativity across cultural groups. Therefore, we tested and discussed psychometric properties and results of convergent validity of the Maternal Positivity-Negativity scale in the methods section of the main quantitative study, and not within the main study results.

## Materials and Methods

### Participants

Data from 758 mothers native of their countries and their children between 8 and 12 years old were collected in Poland, the Netherlands, and Turkey (for details of demographic variables per country, see Table 1).

### Measures

The same novel emic scale was used in all cultural groups to assess maternal positivity and negativity toward the child. Two well-known etic instruments were used to measure child attachment security and insecurity. The factor structure of the measures and their measurement invariance across cultural groups were tested before proceeding with cross-cultural hypotheses tested using exploratory and confirmatory factor analyses (Supplementary Material B).

#### Maternal Positivity and Negativity Toward a Child

**FIGURE 1:**
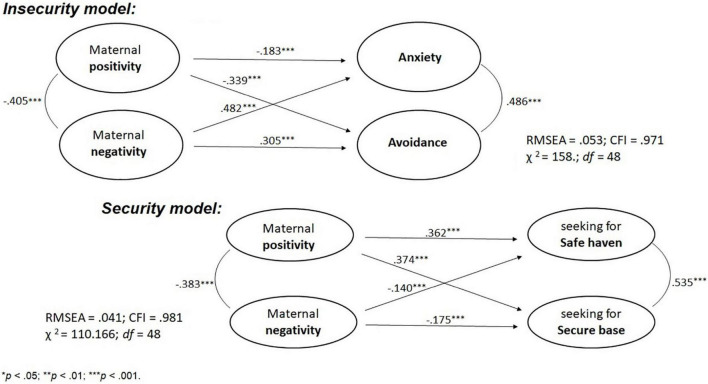
Relations between maternal positivity, negativity and child’s attachment avoidance and anxiety (Insecurity model) and seeking the mother as a secure base and a safe haven (Security model) in the total sample. All relations are significant at the level of ^***^p < 0.001.

**FIGURE 2:**
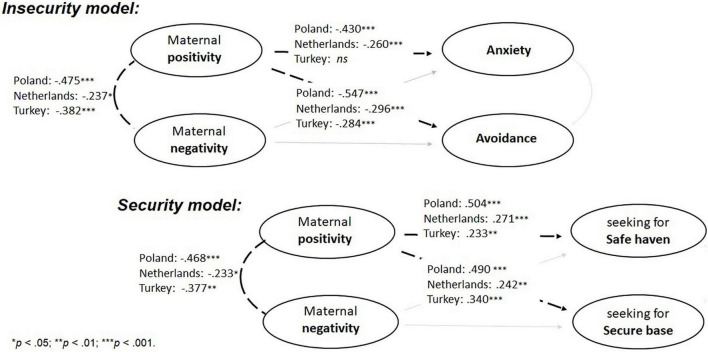
Results of analyses testing cultural group as moderator of relations between maternal positivity, negativity and child’s attachment avoidance and anxiety (Insecurity model) and seeking the mother as a secure base and a safe haven (Security model). All relations are significant at the levels of: *p < 0.05; ^**^p < 0.01; ^***^p < 0.001.

The Maternal Positivity-Negativity scale consisted of 21 statements (see Supplementary Material A: Table 2). Factor analysis of the scale confirmed a two factor structure composed of Positivity and Negativity subscales (see details in Supplementary Material B). Seven items assessed maternal positivity, whereas 10 items assessed maternal negativity. Alpha-based/omega-based reliability coefficients in cultural groups ranged from 0.838/0.789 to 0.900/0.881 (Dutch and Turkish groups, respectively) and from 0.772/0.636 to 0.857/0.721 (Dutch and Turkish groups, respectively) for Positivity and Negativity, respectively. Partial metric invariance was evidenced. Covariance between positivity and negativity was negative and moderate across samples (see Figure 1) and moderated by culture (see Figure 2).

To test the convergent validity of the Maternal Positivity-Negativity scale, we analyzed its relations to other parenting and relationship quality constructs measured by well-known standardized instruments such as: the Network of Relationships Inventory—Behavioral Systems Version (Furman and Buhrmester, 2009), assessing conflict, criticism, companionship, and antagonism, and the Psychological Control Scale (Barber, 1996) measuring psychological control (with a majority of items related to intrusiveness). Our positivity scale was moderately correlated with companionship (0.397; *p* < 0.001) and with psychological control (−0.387; *p* < 0.001). It was also weakly correlated with criticism, conflict, and antagonism (−0.237, −0.174, and −0.202, respectively; *p*’s < 0.001). The negativity scale was moderately correlated with psychological control (0.507; *p* < 0.001), criticism, conflict, and antagonism (0.354, 0.411, and 0.369, respectively; *p*’s < 0.001). Correlations varied across cultural groups with the lowest correlations seen in the Dutch group.

#### Positivity-Negativity Ratio

The Positivity-Negativity ratio (P-N ratio) was calculated based on our Positivity and Negativity measures by subtracting negativity from positivity mean scores for each mother. Scores below zero indicate the prevalence of greater negativity over positivity, whereas scores above zero indicate the prevalence of greater positivity over negativity. The value of the score indicates the size of the difference between positivity and negativity for each mother. The mean level (and *SD*) of the P-N ratio was 1.670 (1.036) in the total sample, and 1.499 (1.045), 1.615 (0.822), and 1.901 (1.173) in the Polish, Dutch, and Turkish groups, respectively. Only 50 mothers (18 in Poland, 5 in the Netherlands, and 27 in Turkey) reported more negativity than positivity in their relationship with their children.

#### Attachment Security

Two subscales of the Network of Relationships Inventory—Behavioral Systems Version (Furman and Buhrmester, 2009; see also the Maternal positivity and negativity toward a child scale for details) were used to assess attachment security of children. Three items assessed the extent to which children treat their mother as a safe haven in times of distress (e.g., “How much do you seek out your mother when you’re upset?”). Another three items assessed how much children are treating the mother as available to provide a secure base for exploration and acting in the external world (e.g., “How much does your mother encourage you to pursue your goals and future plans?”). Results of factor analysis confirmed a two-factor structure of the scale. The Likert scale ranged from (1) “never” to (5) “always.” Alpha-based/omega-based reliabilities in cultural groups ranged from 0.799/0.817 to 0.873/0.875 (Dutch and Polish groups, respectively) and from 0.757/0.768 to 0.774/0.777 (Turkish and Polish groups, respectively) for the safe haven and the secure base, respectively. Covariance between latent factors was not moderated by cultural group and was moderate in the total sample and across groups (Figures 1, 2). Results of analyses supported partial metric invariance of the scale (Supplementary Material B).

#### Attachment Insecurity

The Experiences in Close Relationships Revised—Child version scale (ECR-RC; Brenning et al., 2011) was used to assess two insecurity dimensions. Factorial analyses revealed that half of the items did not have acceptable size or/and the sign of factor loading within cultural groups and in the pooled sample. Thus these problematic items were excluded from further analyses. The child anxiety about the relationship with their mother was assessed with 10 items related to fears of being rejected, not accepted or abandoned by the mother (e.g., “When I show my mother I love her, I’m afraid she doesn’t love me just as much”). Another eight (key-reversed) items assessed avoidance of closeness with the mother (e.g., “I don’t like telling my mother how I feel deep down inside”). Children were asked to respond using a Likert scale ranging from (1) “definitely not” to (5) “definitely yes.” Alpha-based/omega-based reliabilities in cultural groups ranged from 0.863/0.838 to 0.932/0.928 (Dutch and Polish groups, respectively) and from 0.856/0.827 to 0.905/0.899 (Dutch and Polish groups, respectively) for anxiety and avoidance, respectively. Partial metric invariance was evidenced (Supplementary Material B). Covariance between avoidance and anxiety factors was moderate in the total sample and across groups and was not moderated by cultural group (Figures 1, 2).

### Procedure

Data was collected within the CEE-PaAtt project in Poland, the Netherlands, and Turkey, aiming to characterize how maternal parenting relates to the attachment quality of middle childhood-aged children in a culturally sensitive way (see text footnote 1). To this end, in the qualitative stage of the project we carried out interviews with mothers (*n* = 30 in each country) in their national languages and in their cities of residence. The conceptual content analysis of interviews (audio-recorded and transcribed verbatim) with blind coding was carried out twice, by two national collaborators in each country and afterward in a group of all cross-national collaborators. Categories found by each coder were compared until agreement was achieved, first within and then across cultural groups. Based on cross-cultural comparisons, less and more culture-specific items were developed, tested, and used in all groups in the quantitative study (*emic* item set). This strategy for scale development can be contrasted with an etic approach in which well-known scales are developed in one (usually Western) culture and transmitted and applied in other cultures.

At the quantitative stage of the CEE-PaAtt project, we used both emic items of maternal positivity-negativity and well-known etic parenting and attachment scales, developed in Western cultural contexts, to assess parenting and attachment. Mothers and their children (250 dyads) were randomly recruited in each country by professional companies experienced in sampling for social surveys. Families were invited to a focus group meeting and asked to fill out a set of the same questionnaires. Mothers answered a set of questions about attachment-related parenting and their own attachment, while children reported on their own attachment and answered questions assessing their general functioning. The study was approved by the ethics committee of Kazimierz Wielki University. Children received a small gift at the end of the survey. Members of each national team were socialized and living in one of these countries and with their teams served as the point of reference for discussions about cross-cultural differences.

## Results of the Main Study

### Preliminary Analyses

Most correlations between variables in our study (see Supplementary Material C) were moderate or close to moderate according to the Cohen’s guidelines (Cohen, 1992). Correlations between security and insecurity indicators were negative and small for the anxiety-security relationship and moderate for the avoidance-security relationship, supporting our notion that both are different attachment constructs. The positivity-to-negativity ratio was more strongly related to insecurity dimensions than separate scores of positivity and negativity. Positivity, rather than negativity, was more strongly associated with security dimensions (positive relation) and avoidance (negative relation). A stronger relationship was also found for negativity-anxiety than for positivity-anxiety relations.

### Main Statistical Analyses

We computed structural equation modeling (SEM) and multiple regression analyses to test our hypotheses. The nationality of mothers was introduced to our models as a moderator to test culture-related hypotheses.

First, we ran two SEM models. In both models, maternal positivity and negativity were tested to predict child attachment avoidance and anxiety (Insecurity model) and child attachment security indicators of safe haven and secure base (Security model). Security and Insecurity models were tested separately as we aimed to analyze relations between positivity, negativity and child attachment security as well as insecurity without controlling for their mutual effects on each other. That might have resulted in a lack of significant predictors of security or insecurity indicators in our study making it impossible to draw conclusions about parenting correlates for child security and insecurity, which may be targeted separately in practical interventions.

The predictive power of maternal negativity was tested against the predictive power of maternal positivity on child attachment in both models in the total sample as well as in each cultural group to test Hypothesis 1. Next, the effect of maternal negativity on child avoidance was tested against the effect of maternal negativity on child anxiety to test Hypothesis 2. Even though we did not expect differences in the effects of parenting on security dimensions we additionally tested whether positivity and negativity, each individually predict safe haven and secure base with similar strength as part of our test of Hypothesis 2. In all comparative analyses testing our hypotheses the relative strength of the compared paths was tested by constraining a particular path equal within or across groups and assessing the significance of the change in the model fit parameters (Δχ^2^). Multigroup SEM analyses were used to test hypotheses addressing the moderation effects of the country denomination. A Satorra-Bentler correction was used to adjust for multivariate non-normal data distribution.

In the second part of our results, the four multiple regression models explaining each of four attachment dimensions were tested. Due to a collinearity problem caused by testing interaction terms with a continuous moderator using SEM analyses (not sufficiently mitigated by orthogonalization) and inadequacy of distinguishing observable indicators from the positivity-negativity subtraction outcome (positivity-to-negativity ratio) we tested Hypothesis 3 and Hypothesis 4 using multiple regression analyses. First, child attachment dimensions were regressed on culture, maternal positivity-to-negativity ratio (P-N ratio) testing Hypothesis 3, and their interaction (P-N ratio*Culture interaction) assessing moderation effect of culture to partially test Hypothesis 6 (Positivity-to-Negativity ratio section). Then, child attachment was regressed on culture, maternal positivity, maternal negativity, the positivity-negativity interaction term (P*N interaction) to test Hypothesis 4, and their three-way interaction (P*N*Culture interaction) was used to assess maternal positivity and culture moderation effects for Hypothesis 6 (Positivity*Negativity interaction section).

Since the results about the unique effects of positivity and negativity will be reported in the SEM base models testing the first two hypotheses, our description of multiple regression results addressing Hypothesis 4 and partly addressing Hypothesis 6 will be limited to the three-way interaction results. R software (R Core Team, 2017) with “lavaan” package (Rosseel, 2012) and SPSS platform were used for data analyses.

### Comparative Effect of Maternal Positivity and Negativity in Cultural Groups

The structural model of Insecurity in the total sample revealed acceptable fit parameters, RMSEA = 0.053 [0.044; 0.063], CFI = 0.971; χ^2^ = 158.700, *df* = 48, GFI = 0.967; TLI = 0.960. Acceptable fit was also found for the Security model, RMSEA = 0.041 [0.032; 0.051], CFI = 0.981; χ^2^ = 110.166, *df* = 48; GFI = 0.974; TLI = 0.974.

Standardized path coefficients of both models (Figure 1) revealed that maternal positivity was positively related to seeking the mother as a safe haven and treating her as a secure base, and negatively related to the child’s attachment avoidance and anxiety. Furthermore, maternal negativity was positively associated with the child’s attachment avoidance and anxiety, and negatively related to seeking the mother as a safe haven and a secure base.

Results of comparative analyses testing Hypothesis 1, presented in Table 2, revealed two patterns of differences in the effects of maternal negativity vs. positivity. First, maternal negativity was more strongly related to child attachment anxiety than was maternal positivity in the total sample and the Turkish sample, thus supporting Hypothesis 1. Second, not in line with Hypothesis 1, maternal positivity was more strongly related to safe haven and secure base of children than maternal negativity in the total sample as well as in the Polish sample. Maternal positivity was also more strongly related to child avoidance than maternal negativity in the Polish sample. Comparison of relationships between maternal negativity and child insecurity dimensions (Table 2, Hypothesis 2) revealed that negativity was associated with child anxiety more strongly than with child avoidance not supporting Hypothesis 2. Additionally, security dimensions were not differently related to maternal positivity and negativity (Table 2).

Results of multigroup SEM analyses revealed that associations between negativity and attachment were not moderated by culture (χ^2^_*diff*_ = 3.922, *ns* for anxiety; χ^2^_*diff*_ = 4.478, *ns* for avoidance; χ^2^_*diff*_ = 0.443, *ns* for safe haven; and χ^2^_*diff*_ = 0.020, *ns* for secure base), thus not supporting Hypothesis 5. In line with Hypothesis 6, significant moderation effects of culture shown in Figure 2, were apparent for the relationship between maternal positivity and the child’s attachment security and insecurity indicators (χ^2^_*diff*_ = 53.336, *p* < 0.001 for anxiety; χ^2^_*diff*_ = 8.370, *p* < 0.05 for avoidance; χ^2^_*diff*_ = 8.521, *p* < 0.05 for safe haven; and χ^2^_*diff*_ = 6.195, *p* < 0.05 for secure base).

The standardized beta weights of all (in)security indicators in the Polish group were significantly larger than beta weights in the other cultural groups with one exception (Table 3) namely, the relationship between positivity and safe haven was not significantly different in the Dutch than in the Polish group. Furthermore, relationships between positivity and child attachment indicators were not significantly different in the Turkish than in the Dutch groups.

**TABLE 3:** Chi square differences (χ^2^_*diff*_) between: (1) a model with free estimated paths versus (2) a model with two compared paths set equal (positivity-attachment versus negativity-attachment anxiety-negativity/positivity versus avoidance-negativity/positivity; and safe haven-negativity/positivity versus secure base-negativity/positivity) in cultural groups and in the total sample.

Model path	χ^2^_*diff*_ after Positivity—Attachment beta weights set equal between cultural groups		
	Polish (PL)—Turkish (TR)	Polish (PL)—Dutch (NL)	Dutch (NL)—Turkish (TR)
Positivity - > Anxiety	10.145** (PL > TR)	5.623* (PL > NL)	2.150 (NL = TR)
Positivity - > Avoidance	5.904* (PL > TR)	5.725* (PL > NL)	0.147 (NL = TR)
Positivity - > Safe haven	7.980** (PL > TR)	1.543 (PL = NL)	1.610 (NL = TR)
Positivity - > Secure base	5.170* (PL > TR)	4.449* (PL > NL)	0.012 (NL = TR)

*p < 0.05; **p < 0.01.

### The Combined Effect of Parenting on Child Attachment Across Cultural Groups

#### Positivity-to-Negativity Ratio

**FIGURE 3:**
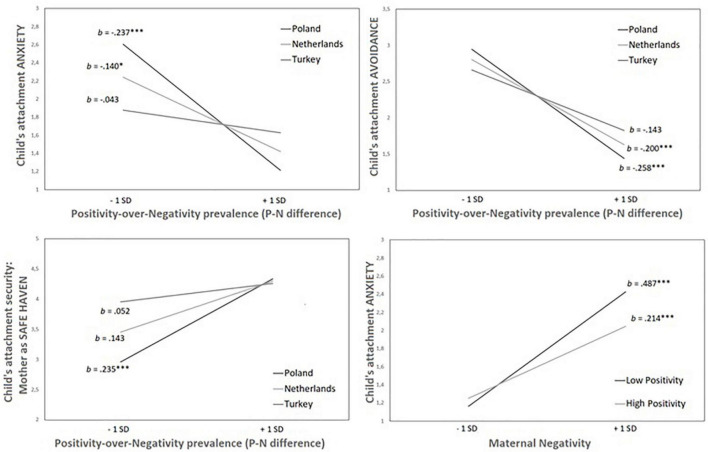
Results of simple slope analyses explaining child’s attachment by: +/− 1SD of maternal positivity-over-negativity prevalence depending on cultural group (upper panels and lower left panel); and low versus high maternal negativity level depending on high versus low maternal positivity level (lower right panel) in the total sample. All relations are significant at the levels of: *p < 0.05; ^***^p < 0.001.

Regression analyses testing Hypothesis 3 revealed a significant P-N ratio*Culture interaction in models explaining child attachment anxiety, avoidance, and safe haven. Culture, P-N ratio, and the interaction of the two cumulatively explained 21% of attachment anxiety variance with half of it accounted for by the interaction term. Maternal P-N ratio (a higher score indicates an advantage of positivity over negativity) was moderately negatively related to child attachment anxiety (*b* = −0.335; *p* < 0.001). The culture was a significant predictor of child attachment anxiety only in interaction with P-N ratio (*b* = 0.097; *p* < 0.001). The maternal P-N ratio predicted child attachment anxiety in the Polish group more strongly than in the Dutch group (Figure 3). The P-N ratio was not associated with child anxiety in the Turkish group.

The same set of predictors explained 19% of the variance in child attachment avoidance. The P-N ratio*culture interaction explained only 0.5% of the variance. The explanative power of the P-N ratio was negative and moderate (*b* = −0.315; *p* < 0.001) whereas the effects of culture (*b* = 0.060; *p* < 0.05) and interaction term (*b* = 0.058; *p* < 0.05) were significant. The relationship between the P-N ratio and avoidance was moderate in the Polish and Dutch groups and insignificant in the Turkish group (Figure 3).

A different pattern of results was observed for the attachment security indicators. Only 15% of the variance of safe haven was explained cumulatively by P-N ratio (*b* = 0.326; *p* < 0.001), culture (*b* = 0.173; *p* < 0.001), and their interaction (*b* = −0.092; *p* < 0.01). The relationship between P-N ratio and safe haven was significant only in the Polish group (Figure 3).

The final analysis of the secure base attachment dimension revealed that only 14% of its variance was explained by P-N ratio and culture. P-N ratio was moderately positively related to secure base (*b* = 0.299; *p* < 0.001), whereas culture revealed a weaker effect (*b* = 0.103 *p* < 0.01). The interaction term of P-N ratio*culture was non-significant. In sum, the results of moderation analyses were in line with Hypothesis 6 revealing that the link between maternal P-N ratio and child attachment was stronger for Polish mothers than for Turkish and Dutch mothers.

#### Positivity*Negativity Interaction

Results revealed that the model explained 21% of the variance of attachment anxiety, whereas 2% was explained by the P*N interaction. In line with Hypothesis 4, the relationship between maternal negativity and child attachment anxiety was stronger when mothers expressed low compared to high levels of positivity (Figure 2). Adding culture to the model did not result in a significant change in variance accounted for, suggesting that this pattern of results is the same across groups. The model predicting child attachment avoidance did not reveal any moderation effect of positivity nor of the interaction or culture and positivity.

## General Discussion

Studies on relationships between daily parental expression of positivity and negativity toward a child and the child’s attachment are scarce and existing results may be biased due to a lack of culturally valid scales directly tapping into parenting behaviors expressing parental positivity and negativity. Thus, we asked mothers in Poland, the Netherlands, and Turkey how they express positive and negative affect toward their children and generated a positivity-negativity scale to collect relevant data. Some maternal behaviors, like ruffling hair, offering something the child likes, or withdrawing child-directed talk, seem to be culturally specific and are hard to find in existing standard measures of negativity and positivity in parent-child relations. Even though our measure taps into similarities and differences in how mothers express positivity and negativity in cultures with varying cultural heritage, our negativity measure partially overlaps with a well-known parenting psychological control scale, especially for intrusive behaviors. This result may indicate that expression of negative affect in mother-to-child relationships occurs largely in the context of psychological control.

Using the positivity-negativity scale, in line with attachment theory, we found that maternal positivity was associated with attachment security, whereas maternal negativity with attachment insecurity of children. However, in the present study we successfully extended these findings through analyses of comparative and combined effects of maternal positivity and negativity on child attachment security and insecurity in various cultural settings. We discuss these main findings from two angles: (1) parenting correlates of attachment in middle childhood and (2) cross cultural variation in the association between maternal positivity/negativity and child attachment using the culture fit hypothesis.

### Relations Between Maternal Positivity, Negativity, and Child Attachment

We propose four major conclusions of our study addressing shared and culturally specific family processes analyzed in our study. First, we found that maternal negativity was associated with children’s attachment insecurity and in particular attachment anxiety (more strongly than their attachment avoidance). We further evidenced that this negativity-anxiety association was even stronger when maternal positivity was low. These preliminary results may be important for parenting practice as they suggest that maternal negativity may be a precursor to child attachment anxiety especially in the context of low-positive mother-child relationships. These results also indicate that maternal positivity may buffer, as a protective factor, the effects of negativity on child attachment anxiety. This conclusion is supported by studies revealing that early experiences of attachment security may be protective factors against later adverse life experiences (Kochanska et al., 2009; Measelle and Ablow, 2017; Oliver and Pike, 2018).

Second, maternal negativity, moderately related to a standardized psychological control scale (Barber, 1996) in our study, was more strongly associated with child attachment anxiety than with avoidance. This preliminary finding is inconsistent with the results of the meta-analysis by Koehn and Kerns (2018), who revealed that maternal intrusiveness (indicating psychological control) predicts children’s attachment avoidance in middle age. Although replication of our findings is needed, it is possible that children’s attachment anxiety and avoidance may be differentially sensitive to various shades of maternal insensitivity delineated differently by intrusiveness and negativity. Negativity and intrusiveness may arise not only based on intrusive parenting practices but also based on maternal rejection or non-intrusive parenting practices, like maternal well-reasoned negative affect disclosure. The causal links between maternal negativity, intrusiveness and child attachment insecurity dimensions are worth further investigation as family-targeted intervention programs could be better tailored to anxious or avoidant children.

Third, we found that children’s attachment avoidance was equally associated with maternal positivity and negativity. This finding does not support the thesis of Baumeister that *bad is stronger than good* (Baumeister et al., 2001; Berlin et al., 2008). Furthermore, child security (safe haven and secure base) was more strongly associated with maternal positivity than negativity, suggesting that *good is stronger than bad* when considering children’s attachment security. This finding is in line with infant studies that reveal the positive effects of maternal sensitivity on child’s attachment security (e.g., Bosmans and Kerns, 2015). Our study is preliminary, however, its results suggest that these findings may be extended beyond early childhood by showing that a link between positivity/sensitivity and security is of major importance in middle childhood as well.

Finally, we tested whether the ratio of greater positivity-to-negativity adds a novel insight into its association with children’s attachment. The overall prevalence of maternal positivity over negativity was comprehensively related to all dimensions of child attachment. It is worth noting that maternal positivity by itself was related to children’s attachment security more than to other children’s attachment dimensions. However, the prevalence of positivity over negativity was more strongly related than positivity to child attachment. Thus, our results preliminarily suggest that the ratio of greater positivity-to-negativity may be an important target of parenting interventions to support children’s emotional regulation capacities. Furthermore, a ratio of greater positivity-to-negativity may be tested further as an explanatory factor of children’s attachment in studies that must, for different reasons, limit their attachment assessment scale to general (in)security. The link between the ratio of greater positivity-to-negativity and security found in our study also relates to the balance theory of marriage (Gottman and Levenson, 2000), showing that a positivity-to-negativity imbalance is related to internalizing problems in various contexts (Zemp et al., 2019).

### Culture-Informed Perspective on Parenting and Child Attachment

From a cross-cultural perspective, our results reveal that the relationship of maternal positivity and the ratio of positivity-to-negativity with all dimensions of children’s attachment were stronger in the Polish sample than in the other two samples. Positivity is traditionally less normative in the Polish cultural context, where it is still a relatively novel strategy used by parents (Dryll, 2013). Complaining about (Wojciszke, 2004) and criticizing children seems to be traditionally normative for the Polish context of socialization and may be treated as indicative of parental negativity. In such a context, explicit expressions of parental positivity may be less frequent and reserved for special occasions. Our preliminary results suggest that the use of maternal positivity may be more beneficial for children’s attachment security in Polish culture than in cultures in which parental positivity expression may be more frequent. In Turkey and the Netherlands, two cultures with indulgent features of childrearing, positivity toward children is more of a norm, thus its link to children’s attachment may be weaker. Indeed, it was weaker in our sample, supporting the culture fit hypothesis.

However, our study also provides results that seem to undermine the culture fit hypothesis. Relationships between maternal negativity and children’s attachment in our study were found to be culturally independent. This finding may question the culture fit hypothesis, according to which parenting negativity should have a stronger effect on child developmental outcomes in cultures in which it is less of a norm (e.g., the Dutch culture) than in cultures in which it is more normative, like in Poland. Struggling with interpretating this result, we propose three explanations and suggestions for future studies. First, we consider the possibility that the culture fit hypothesis is more adequate for testing more general externalizing/internalizing problems (e.g., Lansford et al., 2005), than normative attachment. Second, Güngor and Bornstein (2010) suggest that the father-child rather than the mother-child relationship is sensitive to cultural influences and is more suitable for testing the premises of the culture fit hypothesis. Third and most importantly, we propose that positive parenting behaviors and their developmental outcomes in children may be more sensitive to cultural influences than negative parenting behaviors, thus are worth targeting in future studies. All of our explanations suggest narrowing the extent of generalizability of the culture fit hypothesis in the field of attachment. However, as our study is preliminary, more studies in various cultures are needed to consider these explanations further.

Based on our study, we may preliminarily propose a conclusion that needs further investigation, that *bad parenting* has a bad effect everywhere, whereas *good parenting* brings greater developmental outcomes in some cultural contexts compared to other cultural contexts. It is worth noting that even though this study is preliminary it may fill a gap in the culture fit hypothesis approach by suggesting that developmentally favorable *good parenting* that does not fit the culture may have stronger developmental effects than *good parenting* that fits the culture. Even though the effects of maternal negativity were not moderated by culture in our study, this result is consistent with and adds to previous findings revealing that *bad parenting* is associated with more negative developmental outcomes in parenting-misfit conditions (Lansford et al., 2005; Güngor and Bornstein, 2010).

In line with the culture fit hypothesis, the moderation effect in our study reveals weaker associations between our variables in the culture-fit conditions and stronger associations in the culture-misfit conditions. However, unlike previous studies, the results of our study suggests that parenting positivity rather than negativity may be hypothesized as sensitive to cultural influences. Furthermore, this result may help explain the inconsistency of previous findings on parenting positivity effects on child outcomes. This preliminary result may be useful for clinical practice as it suggests that targeting maternal positivity in parenting programs may have a weaker or stronger effect on children’s attachment from various ecological and cultural backgrounds. In general, our preliminary results suggest that a match between parental behaviors and the cultural views of parenting can act together when shaping child attachment outcomes.

### Impact of the Study

The preliminary nature of the Maternal Positivity-Negativity scale developed for the present study in three national groups is the main limitation of conclusions proposed in our study. This scale is a promising, and its convergent validity was analyzed, thus the results of our study need to be considered as a new contribution to parenting and attachment, however, our findings must be treated with caution and need to be tested further. We do not know whether the same maternal behaviors would be found in another study, and if the results of our study were replicated. A few other limitations of our study should be mentioned: (1) using the nationality of participants (native of their own cultures) as a proxy for cultural group membership; (2) testing the normativity of positivity/negativity in a given cultural context based on assumed but not tested cross-cultural differences in socialization; (3) use of self-report measures that might have activated maternal defensiveness at the implicit level and affected their responses (e.g., Bailey et al., 2016); and (4) maternal parenting and child attachment are shaped through bidirectional mother—child influences that depend not only on cultural influences but also on individual characteristics such as temperament, which was not analyzed in our study. These limitations might affect the strength of relationships found in our study, thus results should be interpreted cautiously.

However, with these limitations noted, our study provides insights into parenting behaviors and directs attention to the importance of a less studied construct: maternal positivity in three different cultures. Furthermore, we provide preliminary statistical evidence informing parenting practice about which parenting dimension (positivity, negativity, or their combination) and which cultural contexts might be targeted in parenting interventions addressing particular aspects of attachment in middle childhood. These findings may also contribute to the attachment field where attachment predictors in middle childhood are understudied and security and insecurity dimensions are rarely studied in combination. Moreover, the analysis of the emotional tone of parent-child relationships beyond parenting dimensions with the use of the new culturally sensitive parenting positivity-negativity measure may add to attachment and parenting studies. With the use of this scale, we show that a positive parent-child affective climate buffers the effects of negativity against child attachment anxiety.

Finally, we provide preliminary evidence informing how cultural contexts may foster or attenuate the sensitivity of child attachment to the effects of maternal positivity and negativity. By doing so, we went beyond testing the cultural generalizability of our findings across cultures but also addressed the culture fit hypothesis in two ways. First, we suggest extending the framework by revealing cultural variation in the misfit between the culture and developmentally favorable behaviors (maternal positivity) that have not been studied before. Second, we ask the question about the generalizability of previous findings revealing that the link between developmentally unfavorable behaviors and culture-(mis)fit is not different across cultures analyzed in our study.

## Data Availability Statement

The raw data supporting the conclusions of this article will be made available by the authors, without undue reservation.

## Ethics Statement

The studies involving human participants were reviewed and approved by the Kazimierz Wielki University. Written informed consent to participate in this study was provided by the participants’ legal guardian/next of kin.

## Author Contributions

KL: scales development, leader of the project and data collection, and writing the manuscript. KG, NS, and ÖA: scales development, data collection, and commenting on the manuscript. WR: scales translation and development. All authors contributed to the article and approved the submitted version.

## Conflict of Interest

The authors declare that the research was conducted in the absence of any commercial or financial relationships that could be construed as a potential conflict of interest.

## Publisher’s Note

All claims expressed in this article are solely those of the authors and do not necessarily represent those of their affiliated organizations, or those of the publisher, the editors and the reviewers. Any product that may be evaluated in this article, or claim that may be made by its manufacturer, is not guaranteed or endorsed by the publisher.

## Footnotes

1
http://attachmentandparenting.com
