# Supplementary material for: Associations Among Maternal Positivity, Negativity and Child Attachment in the Netherlands, Poland, and Turkey
Source: Front Psychol. 2022 May 17;13:820699. doi: 10.3389/fpsyg.2022.820699 (PMC9152534; doi:10.3389/fpsyg.2022.820699)
Supplement: Supplementary file 1 [file Data_Sheet_1.pdf]

Supplementary materials

Supplement A

Results of the Preliminary Qualitative Study Related to Development of the Maternal Positivity-Negativity Scale

Table 1.

Frequency of Positivity and Negativity Behaviors of Mothers in Three Cultural Groups

Positivity/Negativity parenting behaviors of mothers	Cultural group		
	Dutch (*n* =25)	Polish (*n* =30)	Turkish (*n* =24)
**Positivity**			
hugging or cuddling	92% (23)	87% (26)	58% (14)
kissing	44% (11)	53% (16)	46% (11)
stroking their head or ruffling their hair	0% (0)	1% (3)	21% (5)
telling him/her that I love him/her	52% (13)	30% (9)	25% (6)
spending time together	28% (7)	13% (4)	4% (1)
offering him/her something he/she likes	4% (1)	0% (0)	4% (1)
cooking or giving him/her to eat something he/she likes	24% (6)	7% (2)	4% (1)
giving gifts or money	8% (2)	1% (3)	8% (2)
giving him/her more attention and getting involved in his/her activities	24% (6)	30% (9)	21% (5)
showing him/her more warmth in some other way	24% (6)	23% (7)	54% (13)
**Negativity**			
saying	0% (0)	7% (2)	1% (3)
how angry I am	4% (1)	0% (0)	4% (1)
how disappointed I am	0% (0)	3% (1)	0% (0)
what I think and feel	52% (13)	13% (4)	0% (0)
not talking to him/her at all	20% (5)	17% (5)	1% (3)
paying no attention to him/her	4% (1)	3% (1)	0% (0)
being offended	0% (0)	7% (2)	8% (2)
raising my voice or shouting	52% (13)	66% (20)	38% (9)
prohibiting things	0% (0)	60% (18)	0% (0)
pinching or smacking	8% (2)	3% (1)	1% (3)
punishing him/her in some other way	4% (1)	33% (10)	33% (8)

Table 2.

Maternal Positivity-Negativity Scale

(1.) I express **positive feelings towards** my child by …	1<br>strongly disagree	2<br>disagree	3<br>neither agree nor disagree	4<br>agree	5<br>strongly agree
... (1.1) hugging or cuddling.					
…(1.2) kissing.					
... (1.3) stroking their head or ruffling their hair.					
…(1.4) telling him/her that I love him/her.					
…(1.5) spending time together.					
…(1.6) offering him/her something he/she likes.					
…(1.7) cooking or giving him/her to eat something he/she likes.					
...(1.8) giving gifts or money.					
…(1.9) giving him/her more attention and getting involved in his/her activities.					
...(1.10) showing him/her more warmth in some other way.					
(2.) **I express negative feelings towards my child by…**	1<br>strongly disagree	2<br>disagree	3<br>neither agree nor disagree	4<br>agree	5<br>strongly agree
…(2.1) saying …					
… (2.1.1) how angry I am.					
… (2.1.2) how disappointed I am.					
… (2.1.3) what I think and feel.					
… (2.2) not talking to him/her at all.					
… (2.3) paying no attention to him/her.					
… (2.4) being offended.					
... (2.5) raising my voice or shouting.					
…(2.6) prohibiting things.					
... (2.7) pinching or smacking.					
…(2.8) punishing him/her in some other way.					

Supplement B

Results of the Structure of Scales and their Measurement Equivalence across Cultural Groups

Exploratory Factor Analysis (EFA) and Confirmatory Factor Analysis (CFA) were conducted to test the structure of the scales used in our study. First, measurement models were tested separately in cultural groups, later and based on sample-specific results, the suitable measurement model for all samples was tested with the use of CFA. After the structure of the measurement model was considered acceptable, measurement invariance of each scale was tested. Decisions about the invariance were based on one of two criteria, insignificant chi square difference between two models (with and without equality constraints) or difference in CFI of both models below < .01 which is more appropriate for big data sets (Byrne & van de Vijver, 2010). As we did not formulate hypotheses about mean level differences, scalar invariance was not tested and metric invariance was considered to be necessary to be evidenced in our study. Due to the ratio of sample sizes to the number of parameters to be estimated in structural models tested all subscales with more than three items or factors were parceled randomly into three observable indicators of one latent variable (Little et al., 2002). A Satorra-Bentler correction was used to correct for multivariate nonnormal data distribution.

1. Maternal positivity and negativity toward a child

EFA of the *Positivity* subscale revealed a one factor solution in Polish and Turkish samples and supported a two-factorial model in the Dutch sample, where direct expression (e.g. hugging) was loading another factor than indirect feelings expression (e.g., offering something). Nonetheless, CFA supported a one-factorial structure of the scale, RMSEA = .089; CFI = .942, χ^2_{diff} = 116.892; *df* = 39. Measurement invariance analysis revealed that metric invariance was evidenced (χ^2_{diff} = 8.488; *ns*). The structure of *Negativity* was found to be three-factorial with

verbalization, *distance*, and *emotional* factors, RMSEA = .080; CFI = .938, χ^2 = 251.230; *df* = 96. Partial measurement invariance of *Negativity* was established after releasing equality constraints of factor loadings of one item (χ^2_{diff} = 24.576; *ns*). The item set of *Positivity* was randomly parceled into three indicators treated as observable variables of a positive feelings latent factor.

2. Attachment security

This two-factorial attachment security model revealed good model fit, RMSEA = .045; CFI = .992, χ^2 = 36.236; and *df* = 24. Metric equivalence was not supported, nonetheless releasing equality constraints from factor loading of one item indicating Secure base demonstrated partial metric equivalence (χ^2_{diff} = 7.203; *ns*).

3. Attachment insecurity

CFA and EFA analyses of the scale's structure across cultural groups revealed numerous problems with size and sign of factor loadings. Based on that, some items were excluded from further analyses. The final ECR-RC scale analyzed in our study was composed of two factors. Avoidance was composed of one not key-reversed and 8 key-reversed items. The Anxiety factor consisted of 10 not key-reversed items. To test measurement invariance and increase degrees of freedom, these items were randomly packed into three parcels of avoidance and three parcels of anxiety (Little et al. 2002). The model fit was acceptable, RMSEA = .059; CFI = .990, χ^2_{diff} = 29.286; *df* = 8. Partial metric invariance was evidenced after releasing equality constraints of one anxiety indicator and resulted in establishing partial metric equivalence across cultural groups (χ^2_{diff} = 95.487; $p < .001$ and CFI difference below .010).

4. References

Byrne, B. M., & van de Vijver, F. J. R. (2010). Testing for measurement and structural equivalence in large-scale cross-cultural studies: Addressing the issue of nonequivalence.

International Journal of Testing, *10*, 107–132. https://doi.org./110.1080/15305051003637306

Little, T. D., Cunningham, W. A., Shahar, G., & Widaman, K. F. (2002). To Parcel or Not to Parcel: Exploring the Question, Weighing the Merits. *Structural Equation Modeling: A Multidisciplinary Journal*, *9*(2), 151–173. https://doi.org./110.1207/S15328007SEM0902_1

Supplement C

Bivariate Correlations between Study Variables

Parenting and child attachment variables	Parenting			Child Attachment			
	Positivity	Negativity	Pos-Neg ratio	Anxiety	Avoidance	Safe Haven	Secure Base
Positivity							
Negativity	-.308						
Pos-Neg ratio	.776	-.839					
Anxiety	-.328	.388	-.445				
Avoidance	-.388	.313	-.429	.556			
Safe Haven	.374	-.211	.354	-.271	-.494		
Secure Base	.372	-.225	.362	-.166	-.364	.542	

Note. All correlation coefficients are significant at $p < .001$. Pos-Neg ratio – positivity-to-negativity ratio calculated for each mother.
